# Posttraumatic Vernet syndrome without fracture: A case report and short literature review

**DOI:** 10.1097/MD.0000000000027618

**Published:** 2021-10-29

**Authors:** Tamara Braut, Matej Maršić, Iva Ravlić, Diana Maržić, Blažen Marijić, Goran Malvić, Ilinko Vrebac, Marko Velepič

**Affiliations:** a Clinic of Otorhinolaryngology and Head and Neck Surgery, Rijeka University Hospital Center, Rijeka, Croatia; b University of Rijeka, Faculty of Medicine, Rijeka, Croatia.

**Keywords:** case report, cranial nerve traction, dysphagia, dysphonia, posttraumatic Vernet syndrome

## Abstract

**Rationale:**

The aim of this case is to emphasize the need to include nerve traction in the differential diagnosis of nerve deficits associated with Vernet syndrome. This mechanism of injury has been described only once, but must not be overlooked and should be considered and included as a possible cause in diagnostic algorithms.

**Patient concerns:**

A patient presenting with dysphagia, extreme hoarseness, and limited shoulder movement after head injury was admitted to the emergency department.

**Diagnoses:**

Multidisciplinary evaluation was performed, and nerve traction-induced Vernet syndrome was established as a running diagnosis.

**Interventions:**

Intensive swallowing and speech exercises, assisted by a specialist, were performed.

**Outcomes:**

Swallowing and speech exercises significantly and objectively improved the patient's swallowing and voice, with mild hoarseness of voice remaining as the main symptom. Spectral acoustic analysis went from a voice pitch of 163.77 Hz to normal (187.77 Hz), jitter improved from 17.87% to 0.86% and shimmer values decreased from 39.86% to 19.60%. Breathiness during phonation measuring 2.91% was reduced to 1.08% and appropriate average intensity of voice (63.95 dB) was achieved. Initial dysphagia and fluid retention in the right piriform sinus, along with tracheal aspiration, were not observed in control fiberoptic endoscopic evaluation of swallowing.

**Lessons:**

According to our knowledge and literature data, this is the second reported case of posttraumatic Vernet syndrome without radiologically confirmed jugular foramen fracture, induced by nerve traction. Such patients need a prompt multidisciplinary approach in diagnosis and timely posttraumatic rehabilitation therapy for favorable clinical evolution and retrieval of nerve function.

## 1 Introduction

Jugular foramen syndrome (JFS) or Vernet syndrome refers to paralysis of the cranial nerves IX, X, and XI exiting the cranial cavity through the jugular foramen.^[1]^ The causes of the syndrome include various benign and malignant tumors (among the most frequent causes are skull-based metastases), vascular and neurological diseases and lesions, infections, and trauma located in the area of the jugular foramen itself or its vicinity.^[2,3]^

The vast array of possible causes and symptoms, which can be more or less pronounced, as well as the anatomical peculiarities of the region explain the frequently encountered difficulties in the diagnosis of this syndrome.

Trauma-induced JFS is extremely rare, and up to date, some 30 cases of JFS caused by a fracture in the area of the jugular foramen have been reported.^[4,5]^ However, only 1 posttraumatic Vernet case without fracture has been described so far.^[6]^

Therefore, the aim of this case is to emphasize the need to include nerve traction as a possible cause of nerve deficits associated with Vernet syndrome.

## 2 Case report

A 73-year-old woman was admitted to the emergency room after she fell and hit her head against the wall. She complained of extreme voice attenuation, hoarseness, swallowing difficulties, and limited movement of the right shoulder. The patient was hospitalized, and after standard precautionary measures and symptomatic therapy (soft collar, nasogastric tube, anti-edematous therapy, etc), she underwent multidisciplinary diagnostic procedures. The evaluation established impaired right palatopharyngeal reflexes, denivelation of the right palatopharyngeal arch with uvular deviation, and limited elevation of the right shoulder. Flexible nasolaryngoscopy and fiberoptic endoscopic evaluation of swallowing displayed abduction and immobility of the right vocal fold, and fluid retention in the right piriform sinus along with tracheal aspiration.

**Figure 1:**
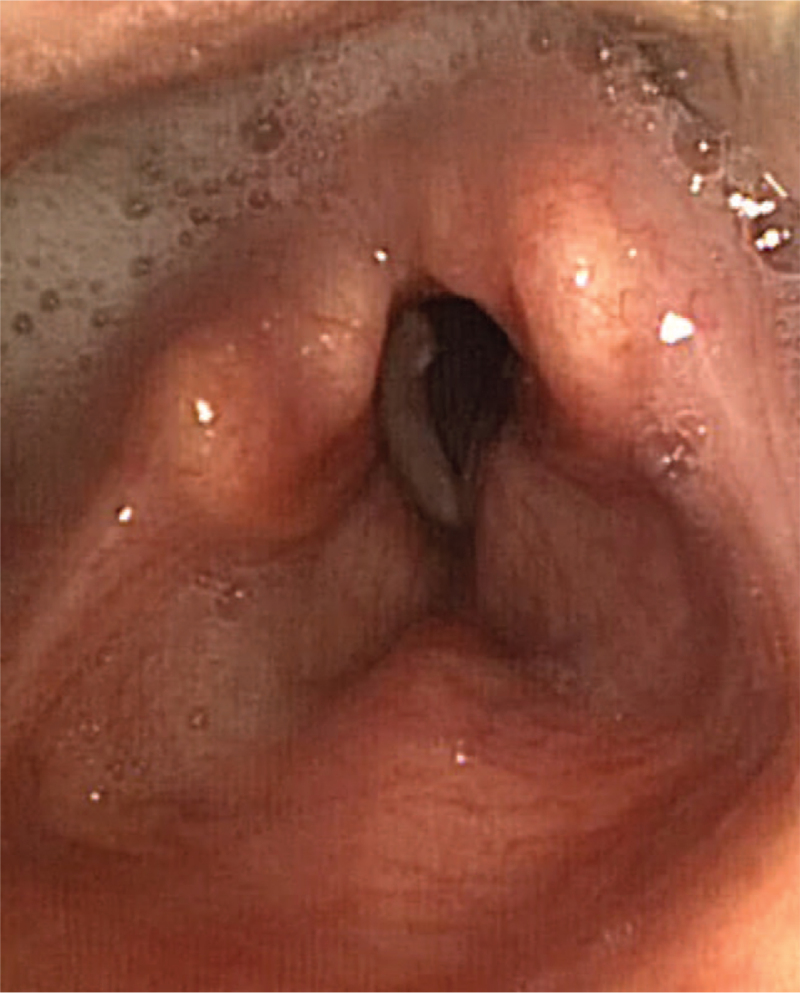
Flexible nasolaryngoscopy showing right vocal fold palsy during phonation, saliva stagnation in the retrocricoid region and right piriform sinus.

Spectral acoustic analysis (Ling Wawes parameters) showed lowered voice pitch (163.77 Hz), jitter (17.87%), and shimmer (39.86%), extreme hoarseness, and increased breathiness during phonation measuring 2.91% (Fig. 1).

**Figure 2:**
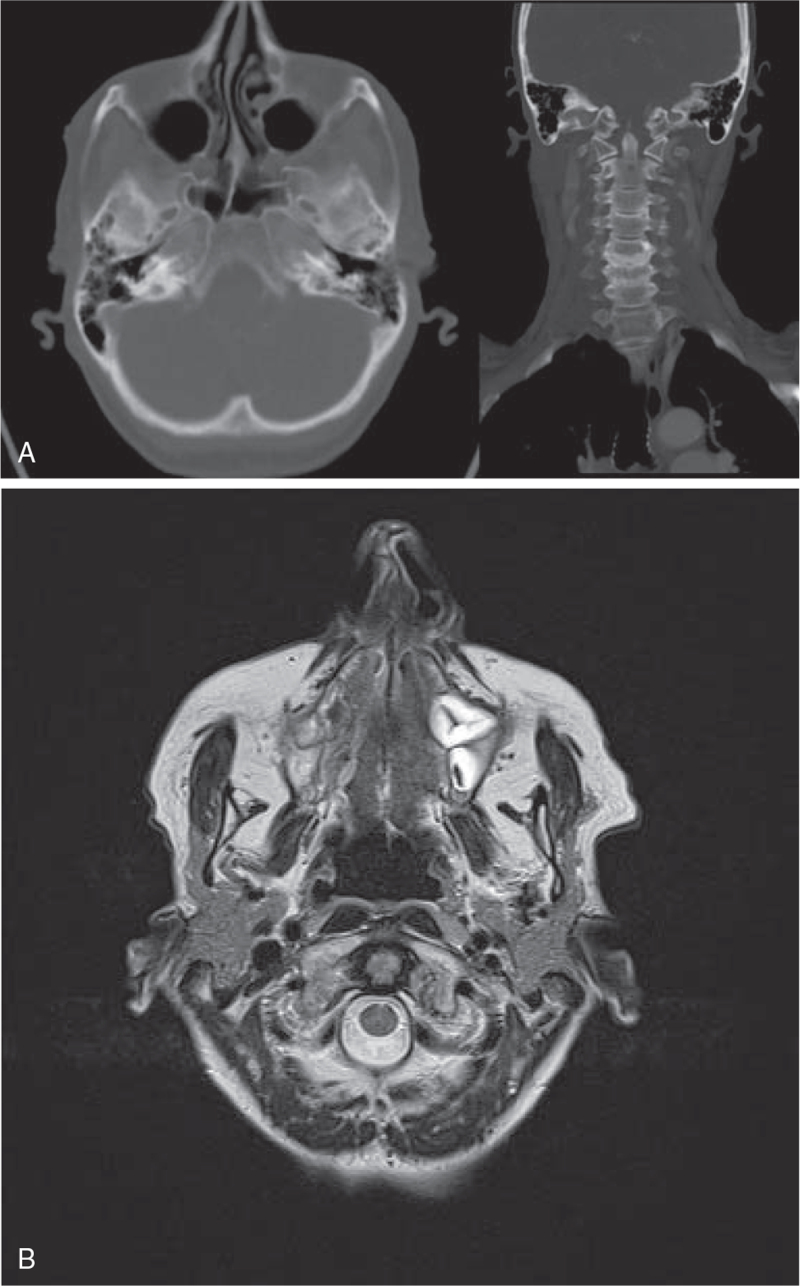
Negative brain radiological imaging (with special attention to the jugular foramen area). (A) Computerized tomography and (B) Magnetic resonance.

Radiological evaluation showed negative brain computed tomography (CT) and magnetic resonance imaging (MRI) scans (Fig. 2a, b). Neuromuscular junction disease was excluded by a negative prostigmine test. An analysis of anti-acetylcholine receptor and anti-muscle specific kinase antibodies also proved to be negative as well as a multidisciplinary specialist assessment.

Given the overall clinical picture, the posttraumatic onset of symptoms, current knowledge of possible causes of Vernet syndrome with the exclusion of tumorous, neurological, vascular, and immunological or infective diseases in the area, the hypothesis of Vernet syndrome caused by traction of the IX, X, and XI cranial nerves were set up.

The patient was prescribed with intensive rehabilitation therapy including swallowing and speech exercises assisted by a specialist. In the next couple of months, her symptoms improved significantly, with normalization of swallowing, mild hoarseness of voice remaining as the main symptom.

**Figure 3:**
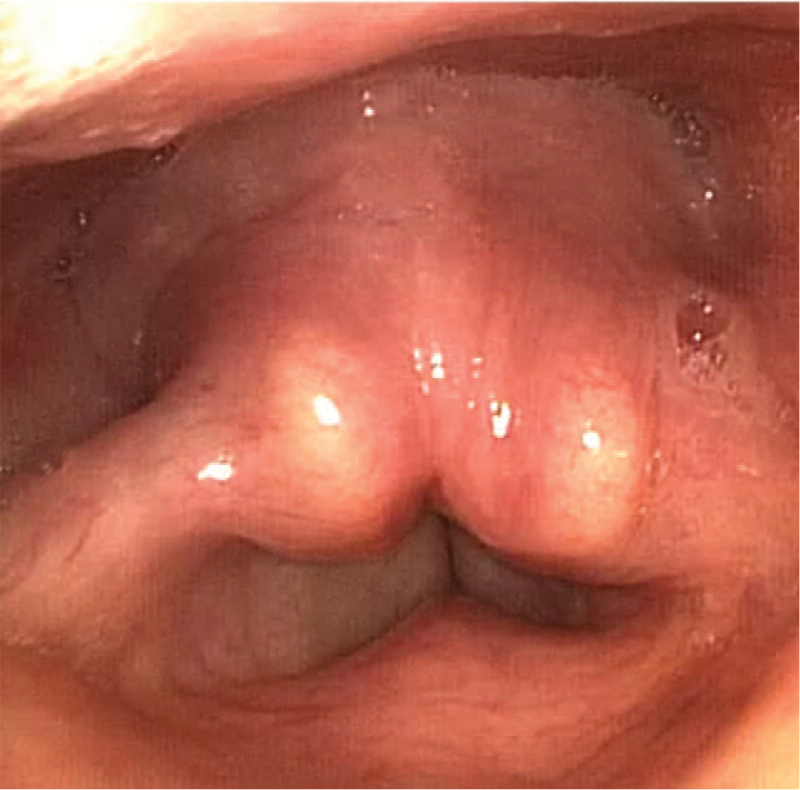
Control flexible nasolaryngoscopy found no fluid retention in the right piriform sinus or tracheal aspiration and satisfactory compensatory movement of the left side with good glottic closure during phonation.

Objective restoration of the palatopharyngeal movements was confirmed with symmetry of the soft palate as well as improvement in trapezoid and sternocleidomastoid muscle movements and shoulder motility, with only a slight denivelation lingering. Control phoniatric evaluation found no fluid retention in the right piriform sinus or tracheal aspiration, the average voice pitch was normal (187.77 Hz), jitter (0.86%), and shimmer values (19.60%) considerably improved with reduced breathiness (1.08%) and appropriate average intensity of 63.95 dB (Fig. 3). The patient was followed up 2 years after the treatment and her voice remained stable.

## 3 Discussion

Vernet syndrome (jugular foramen syndrome) refers to paralysis of the glossopharyngeal nerve (IX), vagus nerve (X), and accessory nerve (XI) traversing the jugular foramen.^[7]^ Symptoms include difficulty swallowing, an absent gag reflex, loss of sensation to the posterior ipsilateral aspect of the tongue, dysfunction of the parotid gland, palatal and pharyngeal paralysis, laryngeal paralysis, muscle wasting, and partial paralysis of the sternocleidomastoid, resulting in the inability to rotate the head or weakness in shrugging the shoulders.^[8,9,10,11]^ Obstruction of the traversing venous sinuses and veins can also occur, resulting in headache and papilledema due to intracranial venous congestion leading to cerebral edema and raised intracranial pressure.^[1]^

Extensive literature research on the topic was performed in PubMed, Medline, Scopus, and Google Scholar databases. According to the literature, the causes of the syndrome can be various benign and malignant tumors, congenital etiologies, inflammatory diseases, vascular and neurological diseases and lesions, infections, and trauma located in the area of the jugular foramen itself or its vicinity.^[2,3]^ Skull-based metastases are the most common cause of Vernet syndrome. Prevalent primary tumors that metastasize to the jugular foramen are breast, lung, kidney, and prostate cancer, as well as melanoma.^[12,13]^ Benign tumors that can lead to Vernet syndrome, include paraganglioma, lower cranial nerve schwannoma, meningioma, aneurysmal bone cyst, neurofibroma, and temporal bone chordoma.^[1,14]^ Most common malignant tumors are squamous cell carcinoma, multiple myeloma, chondrosarcoma, and lymphoma.^[1]^ From congenital etiologies, the primary cholesteatoma can be a reason for developing Vernet syndrome.^[1]^ Giant cell arteritis and Wegener granulomatosis are inflammatory diseases connected with Vernet.^[1]^ Vascular causes of Vernet syndrome can be high jugular bulbs and aneurysms of the extracranial internal carotid artery or vertebral artery, as well as jugular phlebectasia.^[13,15]^ Neurological diseases that can present with JFS symptoms are Guillain–Barrè syndrome, myasthenia gravis, and neuralgic amyotrophy.^[7,16]^ Potential infectious causes of Vernet syndrome are Varicella–Zoster virus infection, Herpes virus infection, *Haemophilus influenzae*, *Neisseria meningitidis*,^[1,7,]^ as well as parapharyngeal abscess, spreading to jugular foramen mostly caused by: *Streptococcus pyogenes*, *Streptococcus pneumoniae*, and *Staphylococcus aureus*. Posttraumatic Vernet syndrome is usually caused by fractures involving the posterior skull base or by penetrating trauma.^[1,5]^ Most cases of fractures in anamnesis have a fall from height while penetrating injuries are usually a consequence of gunshots wounds.^[1,5]^

Diagnostic findings depend on the cause. MRI of the brain with contrast, digital subtraction angiography, CT of the skull base with an angiogram, indirect laryngoscopy, laboratory tests (total blood count, erythrocyte sedimentation rate, C-reactive protein, antinuclear antibodies, extractable-nuclear antigen antibodies, antineutrophil-cytoplasmatic antibodies, serum-protein electrophoresis, Varicella–Zoster virus, and Herpes Simplex Virus (Immunoglobulins M and G), cerebrospinal fluid analysis, nerve conduction studies, and electromyography for the sternocleidomastoid and trapezius muscles can be performed depending upon the possible cause.^[1,17]^

Although there have been several reports on Vernet syndrome caused by fracture, to the best of our knowledge and available literature only 1 case of traumatic JFS without fracture has been reported up to date.^[4,5,6]^ We report the second extremely rare case of posttraumatic Vernet syndrome without radiological confirmation of fracture, with the hypothesis of trauma-induced nerve traction as the most probable cause of the patient's nerve deficits.

Even though we have not confirmed this mechanism radiologically, we have made this conclusion considering the mechanism of injury, sudden onset of symptoms and signs, clinical presentation, and finally, by the exclusion of other diagnoses. Since our patient had no fracture shown by imaging, we can presume that she was exposed to an increase in the angle between the base of the skull and the neck during the fall, resulting in traction and elongation of the nerves that pass through this area. Statistics show that the left vagus nerve is usually wider than the right, and that the right jugular foramen is wider than the left, allowing wider maneuvering space in trauma.^[18]^ According to Hooke law, in the formula Δ*L* = *F*/(*E* · *A*), Δ*L* (the difference in length) is inversely proportional to *A* (the area).^[19]^ From this law, one can conclude that when the external force is applied, the cranial nerves of the left side will stretch in less amount in relation to the nerves of the right. Since nerves tolerate only a certain amount of stretching, a higher stretching rate increases the likelihood of ischemia and nerve lesions and root avulsion.^[20]^ This might also be taken into consideration as an explanation of right-sided nerve deficits observed in our patient.

Treatment of the syndrome depends on its etiology. Possible treatment options include surgery, radiotherapy, embolization, anticoagulants, acyclovir, and steroids, as well as rehabilitation therapy.^[1]^ The prognosis depends on the type of compression at the jugular foramen.^[1]^ The fact that the patient's condition ameliorated after rehabilitation therapy supports the hypothesis of posttraumatic Vernet syndrome due to nerve traction.

Timely diagnostics and intensive rehabilitation therapy enhanced faster recovery and better quality of life in the presented case. Integrated multidisciplinary evaluation and sophisticated diagnostic methods should be advised in any suspicion of posttraumatic Vernet syndrome, especially with negative classical radiological fracture signs. They should be followed by intensive rehabilitation methods that, if administered timely, in an early posttraumatic period like in the aforementioned case, can lead to good symptom improvement, enabling our patients, if not complete recovery, still a good quality of life. This presented case underlines how important it is to include nerve traction as a possible cause in posttraumatic Vernet syndrome. It emphasizes the need for a prompt multidisciplinary approach in diagnosis and timely posttraumatic intensive rehabilitation therapy for a favorable clinical evolution and retrieval of nerve function.

## Author contributions

Tamara Braut, Matej Maršić, and Iva Ravlić are declared as equal first authors, with the rest of the team contributing.

**Conceptualization:** Tamara Braut, Blažen Marijić, Goran Malvić, Marko Velepič.

**Formal analysis:** Diana Maržić.

**Investigation:** Diana Maržić, Ilinko Vrebac.

**Methodology:** Tamara Braut, Blažen Marijić.

**Project administration:** Matej Maršić.

**Software:** Ilinko Vrebac.

**Supervision:** Marko Velepič.

**Visualization:** Blažen Marijić, Goran Malvić.

**Writing – original draft:** Tamara Braut, Matej Maršić, Iva Ravlić.

**Writing – review & editing:** Tamara Braut, Marko Velepič.
